# Targeting Multidrug Resistance With Antimicrobial Peptide-Decorated Nanoparticles and Polymers

**DOI:** 10.3389/fmicb.2022.831655

**Published:** 2022-03-31

**Authors:** Solmaz Maleki Dizaj, Sara Salatin, Khadijeh Khezri, Jyh-Yeuan Lee, Farzaneh Lotfipour

**Affiliations:** ^1^Dental and Periodontal Research Center, Tabriz University of Medical Sciences, Tabriz, Iran; ^2^Department of Dental Biomaterials, Faculty of Dentistry, Tabriz University of Medical Sciences, Tabriz, Iran; ^3^Deputy of Food and Drug Administration, Urmia University of Medical Sciences, Urmia, Iran; ^4^Department of Biochemistry, Microbiology and Immunology, Faculty of Medicine, University of Ottawa, Ottawa, ON, Canada; ^5^Food and Drug Safety Research Center, Faculty of Pharmacy, Tabriz University of Medical Sciences, Tabriz, Iran

**Keywords:** antimicrobial peptides, multidrug resistance, nanoparticles, polymer, peptide

## Abstract

As a category of small peptides frequently found in nature, antimicrobial peptides (AMPs) constitute a major part of the innate immune system of various organisms. Antimicrobial peptides feature various inhibitory effects against fungi, bacteria, viruses, and parasites. Due to the increasing concerns of antibiotic resistance among microorganisms, development of antimicrobial peptides is an emerging tool as a favorable applicability prospect in food, medicine, aquaculture, animal husbandry, and agriculture. This review presents the latest research progress made in the field of antimicrobial peptides, such as their mechanism of action, classification, application status, design techniques, and a review on decoration of nanoparticles and polymers with AMPs that are used in treating multidrug resistance. Lastly, we will highlight recent progress in antiviral peptides to treat emerging viral diseases (e.g., anti-coronavirus peptides) and discuss the outlook of AMP applications.

## Introduction

Antimicrobial resistance impends the fundamental of modern medicine to the continuing hazard from infectious diseases (World Health Organization, 2001). In fact, advancements in modern medicine, such as many medical and surgical procedures, have been facilitated by developing and using clinical classes of antibiotics. Nowadays, the antibiotic era has been threatened due to the alarming rate of multidrug resistance (MDR; Barra et al., 2020; Tuttobene et al., 2021). Furthermore, secondary infections by MDR in complicated conditions like cancers may increase the mortality (Hota et al., 2009; Yan et al., 2020; Hancock et al., 2021). Hence, to address the persistent global issues associated with MDR-related infectious diseases, there is pressing needs to develop new antimicrobial strategies and agents (Ballus et al., 2015; Faya et al., 2018).

Antimicrobial peptides (AMPs), also called host defense peptides (HDPs), are the biomolecules expressed spontaneously by a large array of species throughout the phylogenetic structure (Mahlapuu et al., 2020; Han et al., 2021). AMPs have the enhanced properties of complex functional features, especially antimicrobial, anti-biofilm and anti-inflammatory abilities (Hancock et al., 2021). They are able to combat microbial infections directly, inhibit bacterial activity, and modulate the host immune responses (Drayton et al., 2020). These peptides can be considered as one of promising strategies to MDR problems since they have broad-spectrum activities, multimodal properties, and minimal resistance generation compared to the conventional antibiotics (Deslouches et al., 2020; Tan et al., 2021). Several key problems however remain challenges in the use of AMPs as clinical drugs, including *in vivo* liability, toxicity, and high production costs. Although the efforts to reduce the costs are heavily ongoing; application of alternative production methods, such as biotechnological engineering, bacterial expression systems, or fermentation (Moretta et al., 2021). Furthermore, as the length of peptides directly translates into production cost in solid-phase synthesis; hence, the discovery of ultrashort and/or truncated AMPs with amino acids has been chased (Koo and Seo, 2019). Finally, some modifications are also required to improve antimicrobial activity and to increase the therapeutic potential for next-generation antimicrobial agents *via* chemical or physicochemical alteration of AMPs (Deslouches et al., 2020; Li et al., 2021). Nano delivery systems loaded with AMPs could be promisingly used to enable safe delivery of AMP and decrease their toxic side effects (Tang et al., 2021).

According to its updated version (October 18, 2021), the antimicrobial peptide database (APD3)^1^ has reported a total of 3,283 AMPs (Wang et al., 2016). The commonalities of various types of AMPs are: all AMPs are almost cationic (with an average net charge of 3.32), and the number of their residues of amino acids ranges between 10 and 60 (with an average of 33.26; Malkoski et al., 2001; Schittek et al., 2001). In this review, we will highlight recent updates regarding the classification, potential applications, and mechanism of action of AMPs and the incorporation of AMPs into Nanoparticles and Polymers which have the potential to enhance AMP activity. In addition, we will summarize the application of the AMPs and the latest research progress of using such reagents to treat emerging viral and bacterial diseases, as well as multidrug resistance from the pathogenic microorganisms. Finally, we will provide our perspectives and discuss the outlook of AMP applications in MDR.

## Classification and Mechanism of Action for AMPs

AMPs are made of diversified molecules, which can be classified into four categories on the basis of secondary structural properties: β-sheet, α-helical, loop, and extended peptides (Hancock and Lehrer, 1998; Hamscher et al., 2003), and the common feature among most AMPs is the high content (nearly 50%) of hydrophobic residues and of a net positive charge. Other AMPs belonging to the amphipathic α-helical peptides group include examples, such as LL-37, cecropin, and magainin (Andersson et al., 2003). The β-strand- or β-sheet-like antimicrobial peptides, such as protegrin and the defensin family, include the structural features of two or more disulfide bridges to stabilize their conformations (Nguyen et al., 2011). The highly stable peptides constitute the less frequent group of antimicrobial peptides, which present hairpin-like loop structures interconnected *via* a minimum of one disulfide bridge (Wu and Hancock, 1999), for example, tachyplesins (Xu et al., 2008) or dodecapeptide (Dings et al., 2013). In addition, the extended AMPs often feature a higher content of tryptophan, proline, histidine, and arginine residues. As a result, these peptides can develop unusual secondary structures, such as the well-studied bactenecins and indolicidin (Frank et al., 1990).

Recently, many attempts have been made to develop and identify the synthetic AMPs as well (Fjell et al., 2012). The logic behind this is to shorten the size and thus optimize for bioavailability, metabolic stability, and the issues related to immunogenicity and safety for clinical trials. In addition, shorter sequences are cost-effective. Strøm et al. (2003) demonstrated the minimal length of cationic AMPs that are critical for antimicrobial activities. They showed that a varied content of charge and of bulk and lipophilicity are necessary to produce an active antibacterial peptide. For instance, a synthetic cationic tripeptide containing of one modified tryptophan edged by two arginine residues showed to eradicate gram-positive bacteria through pore formation with a MIC range of 2–4 μg/ml. As another example, HB1345 is a hexameric lipopeptides is administrated topically for the removal of broad skin infections. The MIC value of HB1345 is reported to be 1–2 μg/ml against Propionibacterium acnes (Koo and Seo, 2019). Subsequent introduction of unnatural amino acids, modification or replacement of peptide bonds within an antimicrobial peptide sequence made it possible to reach higher activity levels (Thaker et al., 2011).

AMPs are a class of antimicrobial agents that are promising to limit the development of antibiotic resistance and act as hopeful new weapons to defend against microbial pathogens. Additionally, since these agents can interact with human cell membranes, AMPs may be applied as delivery routes for various bioactive compounds, as well (Guaní-Guerra et al., 2010). While there is a promising prospect for the applications of antimicrobial peptides, AMPs are associated with these problems. (i) AMPs can disrupt the eukaryotic cell membrane; (ii) AMPs may be technically and costly to produce; (iii) Their enzyme stability or pH-sensitive pattern can vary; and (iv) The presence of specific serum and iron may cause reduction of AMP activities. To address these concerns, an ideal next-generation AMP should have these characteristics: (a) targeted action for greater antimicrobial potency; (b) minimal-to-no toxicity for the cell membranes; (c) good stability against proteases or environmental changes, such as pH or temperature; (d) slight serum binding ability; and (f) easy access and low production cost (Brogden and Brogden, 2011; Li et al., 2017). Hence, there is a need to engineer and design AMPs in order to achieve their preferred effects. The rational strategy of antibacterial peptides will consider these five properties: secondary structure, chain length, hydrophobicity, amphiphilicity, and net charge (Lei et al., 2021).

In addition, functionalized AMPs can be achieved by making peptide conjugates. Using various coupling approaches, it is very common to modify at the N-terminal end of the alpha-amino group with a carboxyl group yielding to an amide bond, an approach that is often done when the peptide is still attached to the solid support by employing standard reagents used in solid-phase peptide synthesis (SPPS). Alternatively, the conjugate can be produced in solution using the free peptide if chemo-selective coupling methods is used. The later exploits the activation by thiol-reactive maleimide groups, or amino reactive *N*-hydroxysuccinimides (NHS), on top of procedure, such as copper(I)-catalyzed alkyne-azide cycloaddition (CuAAC), among others (Goodge and Frey, 2020; Huan et al., 2020).

**Figure 1:**
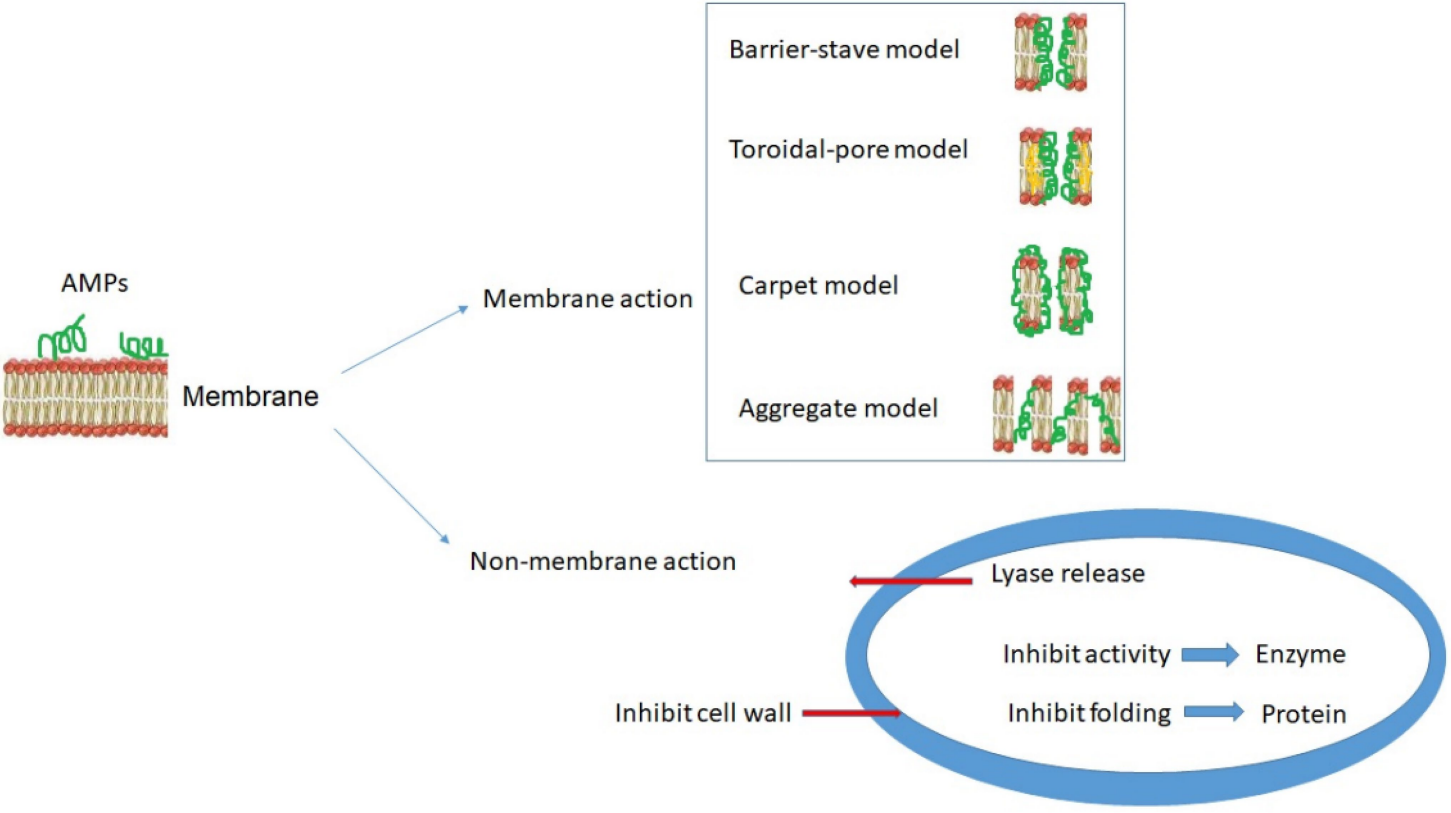
Models of antibacterial mechanisms of antimicrobial peptides (AMPs).

AMPs interact with pathogenic membranes by their surface electrostatic potentials and formation of secondary structures, such that the bacterial membranes can become permeable and eventually destroyed (Da Costa et al., 2015). Understanding the mechanism of AMPs will thus benefit clinical investigations that are needed to understand and evaluate how they work (Schäfer and Wenzel, 2020) and the challenges, such as toxicity of AMPs against host cells (Huang et al., 2010). Due to their cationic and amphiphilic structure, AMPs are believed to interact with negatively charged molecules (such as lipoteichoic acid and lipopolysaccharides) on the microbial outer membrane (Reinhardt and Neundorf, 2016). Hence, the lipid composition of the bacteria has a key role in the sensitivity to the AMPs especially among gram-negative bacteria with significant amounts of both anionic and zwitterionic lipids (Epand and Epand, 2009; Sani and Separovic, 2016). Specific mechanisms that have been observed or proposed include the breakdown of the membrane potential or the leakage of intracellular components, subsequently leading to cell death. So far, as illustrated in Figure 1, the toroidal-pore, the barrel-stave, the carpet-like, and the aggregate models are proposed for membrane-dependent action of AMPs (Brogden, 2005; Schäfer and Wenzel, 2020). The new findings of the toroidal-pore model are showed as an efficient antimicrobial mechanism through the accumulation of peptides on the membrane surface. This aggregation of peptides on the membrane surface and interaction through phospholipid head groups causes the membrane to bend continuously through the pores. Finally, the formation of large diameter pores causes the cell membrane disintegration in microbial pathogens (Matsuzaki et al., 1996; Yang et al., 2001).

The barrel-stave model on the other hand is a two-step process. In this model, the hydrophobic surface of the peptides connected to the microbial membrane and then the peptides enter the hydrophobic region. At the same time, the hydrophilic surface of the peptides tends inward to form a hydrophilic core (Ehrenstein and Lecar, 1977; Di Luca et al., 2014; Kang et al., 2014). Lastly, in the carpet-like model, peptides surround the entire surface area of the bacterial membrane like a carpet. Then, peptides bound to the surface of the bacterial membrane facilitate permeability, and subsequently, the microbial membrane is disrupted by peptides through the mechanism of micelle formation (Pouny et al., 1992; Bechinger, 1999; Fernandez et al., 2012). In addition to membrane permeability processes, AMPs can target microbial pathogenesis through membrane-independent mechanisms (Figure 1, lower panel), including intracellular targets through the inhibition of cell wall synthesis, disruption of DNA, RNA and protein synthesis, and inactivation of relevant enzymes (Brötz et al., 1998; Patrzykat et al., 2002; Kavanagh and Dowd, 2004). Schäfer and Wenzel (2020) recently highlighted several *in vivo* tools allowing mechanistic investigations of both membrane-active AMPs and AMPs with other targets.

## Incorporation of AMPs Into Nanoparticles and Polymers

Recent advancement in nanotechnology allows research to minimize the adverse effects of natural or synthetic AMPs. Nanotechnology, an emerging field of science and engineering over the past few decades, studies designing, characterization, production, and applications of the materials, structures, tools, and systems at the nanoscale (Robles-García et al., 2016), and in particular, biomedicine benefits greatly from nanotechnology (Hoyt and Mason, 2008; Rangayasami et al., 2021). One of the major goals of nanotechnology in designing nanocarriers is to promote biomaterial delivery that can increase the effectiveness of treatment, minimize side effects, provide controlled pharmacokinetic properties, and deliver directly to the target organ with, for example, the encapsulated peptides that are protected against degradation and can reduce toxicity. For this, various nanomaterials, such as polymers, liposomes, hydrogels, and self-assembly systems, are consisted of surfactants, block (Co) and (micro) polymers of polar lipid polymers, or inorganic nanoparticles/nanomaterials that each system-specific, have been used as delivery vehicles not only for peptide transport (Weaver et al., 2021) but also for gene therapy (Bai et al., 2019), cancer therapy (Dong et al., 2019) and drug administration (Martin-Serrano et al., 2019). Drug delivery systems like grafting of AMPs into nanoparticles and polymers at the first glance could be probably sound costly or cause the additional complexity of synthesis, cost, and regulatory hurdles, but they cannot be ignored in some cases like topical or oral peptide delivery. On the other hand, by increasing the drug stability and availability, industries are increasingly more convinced to use them (Cheng et al., 2012). Table 1 summarizes some of studies in regard of different types of nanomaterials decoration by AMPs and the main findings.

**Table 1:** Different types of nanomaterials incorporated with AMPs.

Nanomaterial type	Description	Example for AMPs conjugated nanomaterilas	Main findings	References
Polymeric nanoparticles	They are nanoparticles made of polymer materials with a structural flexibility.	Polymeric nanoparticles (PLGA)- AMPs (LL-37)	PLGA-LL37 NP significantly accelerated wound healing compared to PLGA or LL37 administration alone.	Chereddy et al., 2014
Metal and metal oxide nanoparticles	Metal and their oxide form nanoparticles	AMPs (peptide LLKKK-18)-silver nanoparticles	AMPs-silver nanoparticles showed potent effects in killing mycobacteria They displayed no cytotoxicity or DNA damage on the macrophages from the infected subjects Macrophages actively endocytosed fluorescein isothiocyanate-labeled conjugates causing in nitric oxide independent intracellular killing of mycobacteria.	Mohanty et al., 2013
Micelles	Micelles are described as nano-based drug delivery methods particularly for medicines that cannot be easily soluble in aqueous media.	Micelles (cationic ammonium surfactants)- AMPs (amide moieties)	Micelles-AMPs can self-assemble showed enhanced antimicrobial functionality.	Zhou et al., 2016; Williams et al., 2012
		Micelles (stabilized phospholipid)- AMPs (KSLW-SSM)	Had no effect on bacterial load in stressed mice.	
Liposomes	Liposomes are spherical vesicles featuring the lipid bilayers.	Liposome (Pro-lipo H)-encapsulated AMPs (nisin Z)	In comparison of free nisin, the liposome-based formulation showed higher entrapment efficiency, decreased susceptibility to destabilization and improved efficacy.	Benech et al., 2002
Nanofibers	Nanofibers (fibers with diameters smaller than 100 nm), made of polymers	Incorporating AMPs (plantaricin 423 and ST4SA) into poly D, L-lactide and poly ethylene oxide nanofibers	Combining AMPs and nanofibers thus offers the potential applications in the pharmaceutical and food industries to control microbial infection.	Heunis et al., 2011
Mesoporous nanoparticles	Mesoporous nanoparticles are the materials featuring pore sizes ranging from 2 to 50 nm with honeycomb porous structures.	Silica particles loaded with AMP (LL-37)	Given their higher negative surface charges, a more resilient LL-37 surface coating is formed by the nonporous silica particles, which improves the antibacterial effect and shows particle-mediated membrane interactions.	Braun et al., 2016
Carbon nanotubes	Carbon nanotubes are the rolling carbon-based sheets into seamless cylindrical tubular shapes.	SWCNTs-Ag functionalized *via* AMPs (TP359)	The prepared material showed a decreased cytotoxicity, additive antimicrobial behavior.	Chaudhari et al., 2016
Hydrogels	A cross-linked hydrophilic polymer that does not dissolve in water containing porous 3D matrices.	FEFEFKFK peptide-based hydrogels	The prepared hydrogel retained the drug for a longer time as a platform for combinatory therapy for topical administration.	Tang et al., 2014
		Glutamic acid-alanine-lysine-alanine self-assemble and generate stable hydrogels	The prepared hydrogel improved epithelial regeneration and vascular density, fight infections and promote the skin regeneration.	Rice et al., 2013
		Gelatin methacryloyl and methacryloyl-biopolymer hydrogels-AMP (Tet213)	The subcutaneous implantation of the prepared hydrogels in rats established the biocompatibility and biodegradation and promoted healing of chronic wounds *in vivo*.	Annabi et al., 2017
Dendrimers	Polymers having unique molecular designs with a high degree of molecular uniformity, low polydispersity	Carbosilane dendron [MalG2(S(CH_2_)2 N + Me_2_ḤCl)]—Peptide (H-CRKWVWWRNR) nanoconjugates	Dendron–peptide nanoconjugates passed through the membrane, caused morphological damage as well as deteriorated the cellular integrity of the membrane.	

### AMP-Polymer Conjugates

Polymers, including polyethylene glycol (PEG), poly Lactic-co-glycolic acid (PLGA), chitosan, poly-L lysine (PLL), and hyper branched polyglycerol (HPG), are broadly employed in AMP drug delivery, in which they act as the agents carrying drugs across cellular membranes and disintegrate when reaching their specific target inside the cells (Zhang et al., 2019; Zhen et al., 2019). These polymers have also been employed in AMP delivery, protecting these peptides from degradation and allowing effective delivery (Cui et al., 2021). As a result, the peptides or antibiotics are packaged and delivered to the infected sites, ensuring precise loading on targeted pathogens and safeguarding the peptides against degradation by efflux channels and proteases (Mays, 2020).

Currently, polymer conjugates are used to administer AMP in three novel ways. First, PLGA was developed to administer esculentin-1a-derived AMP in patients with cystic fibrosis with *Pseudomonas aeruginosa* infection and observed improved vehicle transmission and inhibition of bacterial growth inhibition (Casciaro et al., 2019). Secondly, wound healing may be achieved by PLGA conjugates containing covalently-linked growth factors and K4 AMP. This increased sustained release and cell proliferation, and broad-spectrum antimicrobial activity (Vijayan et al., 2019). Lastly, ultrashort AMP was encapsulated in chitosan nanoparticles, which reduced the toxicity compared to free AMP and improved the antibacterial activity compared to chitosan uncharged nanoparticles (Almaaytah et al., 2017).

### Polymeric Nanoparticles and Nanoparticles-AMP Conjugates

Polymeric nanoparticles, nanoparticles made of polymer materials, have shown countless potential in the treatment of a variety of diseases, owing to their structural flexibility (Elsabahy and Wooley, 2012). The polymeric nanoparticles can then be characterized through their capability of protecting the drugs against degradation by enzymes to facilitate drug crossing the cell wall barriers in bacteria and to control the rate of drug release (Vauthier and Bouchemal, 2009; Elsabahy and Wooley, 2012). In addition, many polymeric nanoparticles are bio-degradable and cleared through metabolic pathways in the bodies (Chan et al., 2010). Moreover, to facilitate the local administration and the release rate, the polymeric nanoparticles that are conjugated with peptides have been shown to demonstrate near-first-order or near-zero distribution without burst release; as a result, it has emerged as a promising system of drug delivery to treat local infections (Piotrowska et al., 2018). This was further exemplified by the application of multi-polymers, such as a combined use of chitosan and polyethylene glycol, a procedure that has been shown to decrease the propensity to aggregate and make the nanoparticles more stable and biocompatible (Calvo et al., 1997).

Antimicrobial polymeric nanoparticles have been shown as novel antibiotics to combat the rise of infectious diseases (Lam et al., 2018). This is especially illustrated by using peptides as a main element in such novel supramolecular assemblies for polymeric nanoparticles. The peptide-based nanoparticles can also improve drug delivery into the target cells by binding the nanoparticles to the cell membrane, followed by passive diffusion of drugs and/or nanoparticle phagocytosis (Soppimath et al., 2001; Salatin et al., 2015). Together, it is possible to develop more targeted antimicrobial agents against the infectious calls.

Application of AMPs at the clinical settings has been challenging largely due to their vulnerability to proteolysis or the potential toxicity to the host (Wong et al., 2020). Encapsulating AMPs, including their conjugates in appropriate nanocarriers, provides an opportunity to overcome such advert effect when targeting the site infected by bacteria and keep the peptides from fast degradation (Roy et al., 2011). The AMPs encapsulation has been successfully applied for effective delivery by using a variety of nanoparticle materials (see below), including metallic nanoparticles (MNPs), micelles, liposomes, nanofibers, mesoporous nanoparticles, carbon nanotubes, and hydrogels (Faya et al., 2018).

LL37 belongs to antimicrobial peptides of cathelicidin family that was identified in humans. They are part of the innate immune system and represent the first line of defense against many invading pathogens. Chereddy et al. (2014) prepared LL37 encapsulated in PLGA nanoparticles (chemical encapsulation technique) and they declared that treatment with PLGA-LL37 NP significantly accelerated wound healing compared to PLGA or LL37 administration alone. According to their study, PLGA-LL37 NP improved angiogenesis, significantly upregulated IL-6 and VEGFa expression, and modulated the inflammatory wound response. Also, they showed antimicrobial activity PLGA-LL37 NP on Escherichia coli (Roy et al., 2011).

#### Metal and Metal Oxide Nanoparticles

Gold and silver-derived metallic nanoparticles (MNPs) have been shown to operate as transportation vehicles for AMPs and their conjugates, in which the functionalized nanoparticles are produced by pairing or adhesion of AMPs on the MNP surfaces (Engin and Engin, 2019). These MNPs cause membrane disruption by binding to microbial surfaces through electrostatic interactions due to the large surface area and high surface charges (Burdușel et al., 2018; Alzharani et al., 2021). Considering that most nanoparticles can concentrate in the liver, spleen, and lymph nodes, use of noble metals as resistant compounds to oxidation and erosion is an excellent option for nanoparticle production and toxicity alleviation (Jahangirian et al., 2017). In particular, silver nanoparticles (AgNPs) have shown strong antibiotic properties by disrupting the microbial cell walls and membranes (Yu et al., 2022). It is believed that the silver-based MNPs can attach to the negatively charged microbial membranes, resulting in a greater positive zeta potential and subsequently membrane rupture and permeation (Mi et al., 2018). Interestingly, recent studies by Mohanty et al. (2013) have shown that the synthesized AMPs-silver nanoparticles (synthesized using plant extract and loopful spore methods) are potent in killing mycobacteria, while the conjugates displayed no cytotoxicity or DNA damage on the macrophages from the infected subjects. Macrophages here actively endocytosed fluorescein isothiocyanate-labeled conjugates causing in nitric oxide independent intracellular killing of mycobacteria.

#### Micelles

Micelles were first described as nano-based drug delivery methods in 1980s, particularly for medicines that cannot be easily soluble in aqueous media (Maghsoudnia et al., 2020). Because of their small sizes, micelles can solubilize hydrophobic medicines that target specific sites (Thassu et al., 2007). Micelles are amphipathic, including hydrophilic and hydrophobic blocks with varying solubility proportions in water (Thassu et al., 2007). Using micelles, some cationic AMPs can self-assemble and have been shown to form a comprehensive range of functions and minimal minimum inhibitory concentration (MIC) rates (Zhou et al., 2016).

#### Liposomes

Liposomes are spherical vesicles featuring the lipid bilayers broadly used in drug delivery studies. It has been shown that liposomes can extend the drug half-life, biodegradability and/or biocompatibility, and decrease toxicity, making them very attractive solutions for biomedical studies (Fathi and Oyelere, 2016; Allahou et al., 2021). Recent studies, for example, showed that liposomes enhance the bioactive molecules’ delivery by acting as a circulating micro-reservoir for sustained releases (Karimi et al., 2015). In addition, liposomes are able to encapsulate both hydrophobic and hydrophilic substances by nature, which makes them an ideal nanostructure for amphiphilic compounds’ delivery (Pinilla et al., 2021). A critical challenge however is the premature delivery of the payloads before reaching the target sites (Swaminathan and Ehrhardt, 2012), or the encapsulation efficiency for clinical dosage is low, which requires the use of higher liposome volumes (Swaminathan and Ehrhardt, 2012). Benech et al. (2002) prepared and investigated the liposome-encapsulated nisin (molecular self-assembling method) and discovered that the encapsulation provided higher entrapment efficiency along with decreased susceptibility to destabilization. In comparison of free nisin, the liposome-based formulation showed an improved efficacy against *Listeria monocytogenes*.

#### Nanofibers

Nanofibers (fibers with diameters smaller than 100 nm), made of polymers specially processed to produce filaments, have shown as another promising vehicles for AMPs or AMP conjugates (Subbiah et al., 2005). To synthesize the nanofibers, scientists utilize electrospinning, a process that the electric stress is used to produce charged strands of polymeric mixture (Subbiah et al., 2005). The polymers can be made of natural or artificial polymers, including chitin, chitosan, polyurethane, poly (L-lactic acid), or polyvinyl alcohol (Hu et al., 2014). Because of the high surface-to-volume proportion and the ability to modify their surfaces, nanofibers are excellent for AMP loading and distribution. Heunis et al. (2011) recently demonstrated incorporating AMPs into nanofibers (using electrospinning method) as a new method for delivering the prepared nanofibers had size ranging from 200 to 450 nm and a loading efficiency of 78 percent. AMPs in wound dressings by making bacteriocins stay active after electrospinning the peptides into nanofiber surfaces. Combining AMPs and nanofibers thus offers the potential applications in the pharmaceutical and food industries to control microbial infection.

#### Mesoporous Nanoparticles

Mesoporous nanoparticles are the materials featuring pore sizes ranging from 2 to 50 nm with honeycomb porous structures (Antonelli and Ying, 1996). The mechanism of forming the mesoporous pores is unclear, while it is possible to control the morphology and the orientation of the pores (Ciesla and Schüth, 1999). Silica-based mesoporous materials are the main group of this materials with the outstanding advantage of their biocompatibility, uniformly tunable pore sizes, modifiable particle sizes, high pore volume, and high specific surface area (Kwon et al., 2013). Due to their tendency and selectivity to interact with cell membrane, the use of mesoporous nanoparticles as AMP carriers has been instrumental and effective for both free peptide and peptide-loaded nanoparticles. Recent studies by Braun et al. (2016) showed that the porosity of mesoporous nanoparticles could control AMP loading and release, as well as the antimicrobial activity. Such modification makes the loading for various quantities and molecular shapes of drugs possible. The larger are the pore sizes, the greater the release rates will be and therefore the higher and better antimicrobial action can be gained.

The antibacterial capacity can also be affected by the charge of mesoporous materials (Jia et al., 2012). By testing cationic and anionic non-porous and mesoporous silica particles loaded with AMP (LL-37), the proteolytic activity on LL-37 was better prevented by the anionic mesoporous silica particles. Given their higher negative surface charges, a more resilient LL-37 surface coating is formed by the non-porous silica particles (size range of 294 to 306 nm and a slow release pattern of LL-37), which improves the antibacterial effect and shows particle-mediated membrane interactions (Braun et al., 2016). The challenge is however the cytotoxicity on the erythrocytes by the nanoparticles of positively charged mesoporous silica. There are thus needs to improve such mesoporous nanoparticle strategy, despite its promotion of membrane-disrupting activity. Nevertheless, other strategies, such as bonding (capping) of a peptide onto the external surface of mesoporous silica nanoparticles, have been reported to produce new controlled delivery systems (De La Torre et al., 2014). This advancement in the application of multipurpose peptides for surface functionalization of mesoporous materials can achieve cell-specific targeting of antimicrobial agents (Yu et al., 2020).

#### Carbon Nanotubes

Carbon nanotubes are the rolling carbon-based sheets into seamless cylindrical tubular shapes (Dresselhaus et al., 2000). Besides the improvement of drugs solubility, they can be employed as peptide transporters, near-infrared photothermal agents, vaccine individual gene carriers, or in cancer therapy (Lin et al., 2005). The antibacterial behavior of carbon nanotubes in disinfection of both Gram-negative and Gram-positive bacteria is attributed to the induction of oxidative stress and the physical bactericidal mode of carbon nanotubes that result in damages to their cellular membranes (Niu et al., 2010; Dizaj et al., 2015). Despite their great antimicrobial effects, the problems associated with their use are their low yields, high synthetic energy demands, and high dependency on hydrocarbons predominantly obtained from petroleum (Dizaj et al., 2015). To overcome such technical barriers, functionalization has been shown to resolve these issues. Chaudhari et al. (2016) tested the single-walled carbon nanotubes (SWCNTs) and covalently functionalized *via* AMPs for antimicrobial activity. Their microscopic results showed the peptide was indeed attached to the SWCNTs-Ag. Besides, the results showed decreased cytotoxicity and additive antimicrobial behavior for the functionalized SWCNTs, a promising step toward novel and biologically compatible nanomaterials.

#### Hydrogels

A hydrogel is a cross-linked hydrophilic polymer that does not dissolve in water (Borro et al., 2020). Nanohydrogels are the polymers containing porous 3D matrices. Which makes hydrogels good aqueous absorbers, can maintain oxygen penetration, and prolong drug retention when employed in topical administration (Xia et al., 2013). Two types of artificial hydrogels have been developed. One is a nanocomposite hydrogel, in which nanoparticles are embedded in hydrogel networks, whereas the other is a colloidal hydrogel of nanoparticle, in which nanoparticles are directly employed as crosslinkers to create hydrogel networks (Manconi et al., 2018).

Recently, nanohydrogels have been the focus of hydrogel use in the pharmacological applications, due to their good biocompatibility and their ability to carry AMPs against skin infections. According to the findings of Tang et al. (2014) a self-assembling octapeptide method was used to develop a hydrogel model that could be loaded with active peptides and other synergistic drugs for *in vitro* bioactivity tests. This retained the drug for a longer time (cumulative releases of 68%, 30%, and 39% were observed after 2 h for the 20, 30, and 40 mg L^−1^ samples, respectively.), a promising way for a combinatory therapy for topical administration. Rice et al. (2013) further showed that once the hydrogels for delivering bioactive molecules known as healing accelerators [e.g., nitric oxide (NO)]; it can improve epithelial regeneration and vascular density, fight infections, and ultimately, promote the skin regeneration. In a recent study, Annabi et al. (2017) designed a new biocompatible hydrogel-AMP conjugate using light-induced crosslinking method and abroad-spectrum antimicrobial activity against Gram-positive or Gram-negative bacteria. The subcutaneous implantation of the prepared hydrogels in rats established their biocompatibility and biodegradation and promoted healing of chronic wounds *in vivo*.

#### Dendrimers

Dendrimers signify a class of different polymers having unique molecular designs characterized by their well-defined structure, with a high degree of molecular uniformity, low polydispersity and properties that make them attractive materials for the development of nanomedicines (Samad et al., 2009). The most common dendrimers are polyamidoamine (PAMAM), polypropyleneimine (PPI), poly-l-lysine (PLL), polyglycerol (PG), poly(benzyl ether), carbosilane, or phosphorous dendrimers (Kawano et al., 2020). Nano dendrimers as carriers for AMPs suffer from some basic shortcomings like the limited efficacy, short-lasting bioactivity, and concerns of toxicity as well as high production costs (Kiparissides and Kammona, 2013). Fernandez et al. (2019) recently reported Carbosilane Dendron–Peptide Nanoconjugates with a maleimide group in the focal point that could attach different AMPs containing a cysteine residue in their structure as antimicrobial agents. In this study, the capacity of dendron–peptide nanoconjugates was shown to pass through the membrane, causing morphological damage as well as deteriorating the cellular integrity of the membrane.

#### Antiviral Peptides

In addition to bacteria-based infectious diseases, viruses are known to cause serious diseases that have a severe impact on economy and public health. *Recently, the outbreak of the COVID-19 has* led to a dramatic *loss of* human *life* worldwide. Other long-term threats to human life include HIV, avian influenza virus (AIV), and foot-and-mouth disease virus. Therefore, there is an urgent need to solve the global problems caused by viruses, and in this case antiviral peptides provide new and promising strategies. Antiviral peptides exhibit inhibitory activity against viruses by (1) impeding viral interaction and membrane fusion, (2) disrupting the integrity of the envelope, and (3) preventing the replication of virus (Eftekhari et al., 2019; Jung et al., 2019). For example, AMP Epi-1 has been shown to exert an excellent anti-foot-and-mouth disease virus effect (Huang et al., 2018). Infectious bronchitis is a viral disease caused by infectious bronchitis virus (IBV), and the mortality of chicken embryos was shown to decrease significantly after they were inoculated with swine intestinal AMP (SIAMP)-IBV solution, compared with the IBV infection group. These results indicated that SIAMP can contribute to an inhibition of this virus (Sun et al., 2010). Nowadays, there is a wide variety of anti-HIV peptides, such as defensins, LL-37, caerin 1, siamycin-I, siamycin-II, dermaseptin-S1, dermaseptin-S4, gramicidin D, maximin 3, magainin 2, and RP 71955. Notably, Fuzeon™ (enfuvirtide) is an approved medication to treat HIV patients (Ashkenazi et al., 2011).

*Owing to* the rapid outbreak of COVID-19 worldwide, we will focus our discussion on the use of antiviral peptides against the coronaviruses. Morphologically, coronaviruses (CoVs), a group of coronaviridae, are enveloped viruses containing a single strand of positive-sense RNA genome and a helical symmetry (Franks and Galvin, 2014). CoVs, including SARS-CoV (severe acute respiratory syndrome CoV) and MERS-CoV (Middle East respiratory syndrome coronavirus; Mustafa et al., 2018), and SARS-CoV-2 (the new strain causing COVID-19) have resulted in outbreaks with life-threatening respiratory diseases over the past two decades. CoVs consist of the membrane (M), the envelope (E), the spike (S), and the nucleocapsid (N) proteins (Boas et al., 2019), in which S proteins are responsible for the viral infection. Fusion inhibitory peptides in principle can interact with the S protein to block its folding, thereby preventing viral infection. The S protein of SARS-CoV contains two heptad repeats HR1 and HR2 domains in their S2 subunit. Peptide HR2 and its lipid-binding subunit appear to be same or similar to the near-membrane part of S protein ferredoxin, which inhibit refolding into post-fusion fusion-catalyzing domains (FDs; Du et al., 2009; Park and Gallagher, 2017). Overall, AMPs against coronavirus can be derived from (i) HR1, HR2 and RBD domains of the S protein, (ii) non-structural proteins, and (iii) other AMPs (Mustafa et al., 2018).

The EK1-derived lipopeptide (SLDQINVTFLDLEYEMK KLEEAIKKLEESYIDLKEL), has been shown to have the highest inhibitory activity against the S-mediated fusion capacity of COVID-19 (Xia et al., 2020). The results obtained by protein-peptide docking and homology modeling indicated that temporin has good inhibitory effect on MERS-CoV (Marimuthu et al., 2020). It was found that AMPs (K12 and K29) derived from the SARS-CoV-non-structural protein (nsp10), can suppress the replication of SARS-CoV (Ke et al., 2012). Moreover, the mortality induced by SARS-CoV reduced remarkably in animals after treatment with rhesus theta-defensin 1 (RTD-1), by which the airway immunomodulatory was a possible process responsible for its action (Wohlford-Lenane et al., 2009). Molecular docking has demonstrated that peptides are able to prevent the attachment of COVID-19 to ACE2 (angiotensin-converting enzyme 2) and suppress the entry of COVID-19 into host cells (Souza et al., 2020), albeit the studies are currently limited at the preclinical stage. Nevertheless, with *the antiviral database (AVPdb2)* available and containing collections of antiviral peptides, peptide-based *drug administration via* the nasal route will exhibit an important area of research for AMPs *as* potential *drugs* for the therapy of coronavirus-caused respiratory diseases. Table 2 summarizes some antimicrobial peptides as an antiviral agent.

**Table 2:** Some antimicrobial peptides with described antiviral activity.

AMP name	Description of AMP	Viral diseases inhibited by AMPs	AMP’s mechanism of action	References
Lactoferrin (LF)	A glycoprotein-based AMP that is isolated from several mucosal secretions.	Coronaviruses, hepatitis C virus (HCV), herpes simplex virus (HSV), human immunodeficiency virus (HIV), polio-, and the rotavirus, SARS-CoV, COVID-19	Able to inhibit replication of viruses. Able to increase the host immunity against viral infection, rather than acting against the virus after infection, by preventing virus entry to the host cell, through blocking cellular receptors, or direct binding to the virus particles.	Vorland, 1999; Van Der Strate et al., 2001; Lang et al., 2011
LL-37	A 37- amino acid cationic peptide produced by cleavage of the antimicrobial domain from the hCAP18 protein. The hCAP18 is 18kD precursor protein with a signal peptide, a cathelin-like domain and antimicrobial domain.	Influenza A viruses	The central fragment of human LL-37 is essential for optimal antiviral activity.	Tripathi et al., 2013
Indolicidin	An antimicrobial peptide isolated from neutrophil blood cells of cows.	HIV	Inactivate HIV by binding to the envelope and cracking the membrane through a membrane splitting mechanism, thereby preventing the virus from infecting the host cell.	Robinson Jr et al., 1998
Defensin retrocyclin 2	Small cationic antimicrobial peptides isolated from the leukocytes of rhesus monkeys.	Herpes simplex virus type 2 (HSV-2)	Can bound to HSV-2 glycoprotein B with high affinity so that HSV-2 could not bind to the surface of host cells.	Yasin et al., 2004
Human β-defensin-3 (HβD-3)	The host defense peptide family isolated from human lesional psoriatic scales and cloned from keratinocytes.	HIV	Inhibits HIV replication by acting on entry, reverse transcription, and nuclear import of retroviral DNA.	Zapata et al., 2016
Piscidin-1	A natural polypeptide isolated from mast cells of fish.	Catfish virus (CCV), frog virus 3 (FV3), and HIV-1	Piscidin-1 can directly interact with virus particles and block cell apoptosis induced by PRV.	Noga and Silphaduang, 2003; Lee et al., 2014
Caerin 1.1	A major antimicrobial peptide isolated from the skin of the Australian green tree frog.	Pseudorabies (PRV)	Can destroy the integrity of virus’s particles by forming holes in the membrane.	Pukala et al., 2004; Guo et al., 2018
Porcine β-defensin-2 (pBD-2)	A cationic antibacterial peptide isolated from the leukocytes of rhesus monkeys.	Pseudorabies (PRV)	Able to destroy the viral envelopes and also affects PRV entry into host cells.	Huang et al., 2020
DNBLK1	A synthesized short peptide and contains DLC8 binding domain.	African swine fever virus (ASFV)	Can reduce the infectivity, replication, and production of ASFV, and the inhibition occurs at the early stage of the ASFV infection cycle.	Hernáez et al., 2010
Epinecidin-1 (Epi-1)	Derived from the orange-spotted grouper and belongs to the piscidin peptide family.	Foot-and-mouth disease virus (FMDV) (type O/Taw/97)	By inactivating virus particles and inhibiting virus proliferation.	Huang et al., 2018

## AMPs and Multidrug Resistance Transporters

The drug resistance process is mediated by various mechanisms leads to the failure of the drug-based treatment of numerous infectious diseases. The expansion of multidrug resistance (MDR) in cancer cells is also another serious risk that many anticancer drugs lose their therapeutic activity. One major cause for MDR is the action of multidrug resistance transporters that block unrelated compounds from retention inside the cells (Bolhuis et al., 1997). The mechanistic understanding of MDR transporters is so far elusive and a main subject of pharmacological/pharmaceutical studies.

Screening for the activity of anticarcinogenic substances led to the isolation of numerous resistant strains, which were then characterized as MDR mutants because they exhibited cross-resistance to multiple distinct medicines (Lomovskaya and Lewis, 1992). In general, the known pharmaceutical transporters throughout eukaryotes and prokaryotes have been classified into two groups based on bioenergetic and morphological standards: (i) ATP-binding cassette (ABC) and (ii) secondary drug transporters (Bolhuis et al., 1997).

ABC transporter family characterized by gene sequence and structural resemblances. This protein superfamily is the main group of cell membrane transporters prearranged into seven subfamilies. Many human ABC proteins, including P-glycoprotein (P-gp/ABCB1/MDR1), breast cancer resistance protein (BCRP/ABCG2/ABCP/MXR; Veitch, 2007), and multidrug resistance protein 1 (MRP1/ABCC1) are efflux transporters, and have been known to have critical role in the development of MDR (Zaman et al., 1994; Hee Choi and Yu, 2014). These membrane transporters have the ability to enhance the efflux of chemotherapeutic drugs to reduce the intracellular concentration of drugs, which is one of the most common reasons of MDR. Human ABCB1 transporter was the first recognized ABC transporter of which its overexpression could induce drug resistance of cancer cells to a series chemotherapeutic drugs like paclitaxel, doxorubicin, and vincristine (Teng et al., 2020).

Recently some antimicrobial peptides are reported to have anticancer activity on cancer MDR cells with less toxicity. Due to the hydrophobic and cationic nature of antimicrobial peptides, they could enable special binding to cancer cells with hydrophobic and negatively charged environments. As well as binding to the cell membrane which cause cell death, antimicrobial peptides can also block the interaction between growth factors and their receptors, activating anti-angiogenic effects; inhibiting specific kinase/protease which promote the growth, invasion, and metastasis of tumor; and inhibiting special functional proteins to stop the development of cancer.

Multidrug resistance during malignancy is often triggered by the overexpression of ATP-binding cassette (ABC) transporters. Upregulation of ABC transporters, which operate as efflux pumps for chemotherapeutic medications throughout cancerous cells, may reduce intracellular drug concentrations compared to their healthy cell counterparts. Novel chemotherapeutic medicines based on antibacterial peptides have been studied, and associations with ABC transporters have been observed in several of these compounds. ABCB1-mediated MDR may be reversed by the peptide of XH-14C, which enhanced the intracellular concentration of paclitaxel, a substrate of ABCB1 (Ma et al., 2015; Robey et al., 2018). Teng et al. (2020) reported that the ATPase function of ABCB1 was stimulated by XH-14C, and they demonstrated by molecular dynamics modeling that the XH-14C-ABCB1 combination was in a sustainable binding position. The therapy with XH-14C did not affect the expression rate or localization of ABCB1.

## Current Progress of AMPs in Medical Applications

Many important prospects for the approved AMPs by the US FDA are used in clinical applications, such as ophthalmology, dental, wound healing, and surgical infection (Izadi et al., 2020). Several AMPs have shown tremendous therapeutic potential against various infectious diseases caused by pathogenic bacteria, including peptide ZXR-2 (FKIGGFIKKLWRSLLA) against pathogenic bacteria of dental caries, *Porphyromonas gingivalis*, *Streptococcus sobrinus*, *Streptococcus mutans*, and peptide PAC-113 (Clinical trial identifier: NCT00659971) as effective antimicrobial agents against oral candidiasis (Chen et al., 2017; Sztukowska et al., 2019; Lin et al., 2021). Also, the results of several AMPs studies including *in vivo* and clinical trials have indicated the good therapeutic potential in treating various surgical infections and wound healing. For example, AMP PXL150 was used as an anti-infective agent in burn wounds in mice. Moreover, results of the third phase of clinical trial studies have reported documents of the pharmacological activities of AMP D2A21 as innovative antimicrobial and wound healing agent in the prevention and treatment of burn wound infections (Björn et al., 2015; Thapa et al., 2020). Regarding the field of ophthalmology, with the popularity of contact lenses and the increasing incidence of eye infections caused by various pathogenic bacteria and fungi (such as *Aspergillus* spp., *S. aureus*, *P. aeruginosa*, *Streptococcus pneumoniae*, and *C. albicans*), AMPs, such as Lactoferricin B and Protegrin-1, are able to control the infection effectively (Silva et al., 2013; Khan and Lee, 2020).

Methods to incorporate lipids in medical applications of AMPs have been proposed as part of drug development, including: (1) development of precursors to decrease cytotoxicity and enhance protease stability, (2) the combinational therapy using conventional antibacterial agents and AMPs, (3) designing efficient therapeutic vectors to express AMPs using engineering antimicrobial probiotics, and (4) development of an appropriate expression system using AMPs and more effective drugs. In this regard, we have observed a comprehensive AMP-formulations related to wound healing including nanoparticles, glutinous rice paper capsules, gels, creams, ointments. Moreover, hydrogels in particular demonstrated that loading AMPs in these formulations can now be used therapeutically in the treatment of wound repair (Borro et al., 2020; Thapa et al., 2020). Recent studies by Yang et al. (2001) indicated that the sponges designed with starch, HS-PEG-SH, and AMPs can significantly inhibit the development of bacterial infections (Chu et al., 2019). A variety of techniques have then been developed for improve the targeting mechanism of AMPs. These methods involve graphene, nanotubes, metal nanoparticles, quantum dots, pheromone-labeled AMPs, local environment-triggered AMPs by enzyme precursor drug release system, pH-activated AMPs, etc. (Magana et al., 2020). Various lab-based experimental results showed that fabricating novel hybrid AMPs, such as hybrid peptide PA2 (a *P. aeruginosa*-targeting peptide)—GNU7 (a broad-spectrum AMP) against the *Pseudomonas aeruginosa* OprF protein can be a promising template for managing infectious diseases (Kim et al., 2020). In line with these findings, the use of various antibiotics, such as daptomycin (a lipopeptide), lugdunin, and telavancin (a glycopeptide), as potential antimicrobial candidates for the design of AMPs and medical management of the infectious patients (Durand et al., 2019; Lampejo, 2020).

Table 3 summarizes some completed clinical trials (from https://www.clinicaltrials.gov).

**Table 3:** Clinical trials using AMPs for treatments.

The study	Condition	Interventions	Locations	Status
Clinical and metagenomic investigation of antimicrobial peptide gel in periodontal treatment	Periodontitis	Drug: 0.85% synthetic antimicrobial peptide TAPS-18 Drug: 0.9% normal saline	Faculty of Dentistry, University of Malaya, Kuala Lumpur, Malaysia	Completed
Antimicrobial peptides in periodontitis	Periodontal disease: chronic periodontitis	Other: periodontal smears	Chu de Reims, Reims, France	Completed
Liver-enriched antimicrobial peptide 2	Type 2 diabetes	Biological: liver-enriched antimicrobial peptide 2 Other: placebo	Center for Clinical Metabolic Research, Hellerup, Denmark	Completed
Role of antimicrobial peptides in host defense against vaccinia virus	Atopic dermatitis		National Jewish Health, Denver, CO, United States	Completed
The estrogen impact on overactive bladder syndrome: female pelvic floor microbiomes and antimicrobial peptides	Overactive bladder	Drug: conjugated estrogen	Loyola University Medical Center, Maywood, IL, United States	Completed
First time in man trial for friulimicin B	Community-acquired pneumonia Staphylococcal skin infections	Drug: friulimicin B	Swiss Pharma Contract Ltd., Basel, Switzerland	Completed
Safety of a single dose of 5 mg of hLF1-11 given to autologous haematopoietic stem cell transplant recipients	Hematopoietic stem cell transplantation Bacterial infections and mycoses	Drug: human lactoferrin peptide 1–11	UMC St. Radboud, Nijmegen, Gelderland, Netherlands	Completed
PK/PD of EA-230 during endotoxemia	Endotoxemia Systemic inflammatory response	Drug: EA-230 Drug: endotoxin Drug: placebo	Intensive Care, Research Unit, Radboud University Medical Centre, Nijmegen, Gelderland, Netherlands	Completed
A study to evaluate the microbiology, safety and tolerability of C16G2 dental strip application	Dental caries	Drug: C16G2 strip Other: placebo strip	John F. Pittaway, DMD, Kalispell, MT, United States Jacqueline Kleven, DDS, Bedford, TX, United States Anthony Henegar, DDS, PA, Irving, TX, United States	Completed
A study of DPK-060 to investigate clinical safety and efficacy in patients with acute external otitis	Acute otitis externa	Drug: DPK-060 Drug: placebo for DPK-060 ear drops	ProMore Pharma	Completed
Phase 2B dose-ranging study of PAC113 mouthrinse in HIV seropositive individuals with oral candidiasis	Oral candidiasis	Drug: PAC113	Drug: PAC113	Completed
A study to evaluate safety, tolerability and efficacy of Lytixar™ (LTX-109) on uncomplicated, Gram-positive, skin infection	Gram-positive, skin infections Mild Eczema/Dermatoses Atopic dermatitis	Drug: LTX-109	Debreceni Egyetem Orvos-és Egészségtudományi, Debrecen, Hungary Miskolci Semmelweis Ignác Egészegügyi Központ és, Miskolc, Hungary Pécsi Tudományegyetem általános Orvostudom’nyi Centum, Pécs, Hungary Szeged University Hospital, Szeged, Hungary	Completed
Effects of a 12-week combined exercise program on ghrelins in obese adolescent girls	Ghrelin	Other: combined exercise program Other: control	Pusan National University Yangsan Hospital, Yangsan, Gyeungsangnam-do, South Korea	Completed
Effects of smoking and vitamin D3 on the levels of human cathelicidin peptide LL-37	Periodontitis	Other: LL-37 levels in gingival crevicular fluid Other: serum vitamin D3 levels Other: clinical parameters	-	Completed
DOM-INNATE: study of SGX942 for the treatment of oral mucositis in patients with concomitant chemoradiation therapy for head and neck cancer	Squamous cell carcinoma of the oral cavity and oropharynx Oral mucositis	Drug: SGX942 Drug: placebo	Arizona Clinical Research Center, Tucson, AZ, United States Loma Linda University Health, Loma Linda, CA, United States Pomona Valley Hospital Medical Center, Pomona, CA, United States (and 50 more…)	Active, not recruiting
Study of PXL01 vs. placebo to inhibit adhesion formation after flexor tendon surgery	Surgical adhesions	Drug: PXL01 Drug: placebo	Dept. of Hand Surgery, Aalborg Hospital, Aalborg, Denmark Dept. of Hand Surgery, Odense University Hospital, Odense, Denmark Klinik für Handchirurgie der Herz- und Gefäß-Klinik GmbH, Bad Neustadt, Germany (and 12 more…)	Completed
Pharmacokinetics and safety of POL7080 in patients with renal impairment	Renal impairment	Drug: POL7080	CRS Clinical Research Services Kiel GmbH, Kiel, Germany	Completed
A study of ascending single doses of surotomycin in healthy participants (MK-4261-008)	Clostridium difficile associated diarrhea (CDAD)	Drug: surotomycin Drug: placebo	-	Completed
Trial of iseganan in prevention of ventilator-associated pneumonia	Pneumonia	Drug: iseganan hydrochloride	Barnes-Jewish Hospital, St. Louis, MO, United States	Completed
MSI-78 topical cream vs. oral ofloxacin in the treatment of infected diabetic ulcers	Diabetic foot ulcers	Drug: ofloxacin Drug: MSI-78	Seattle VA Medical Center, Seattle, WA, United States	Completed

AMPs have drawn increased attention due to having high activity among antibiotic-resistant pathogenesis induced by bacteria, fungi, parasites, and various types of cancer, causing disease in animals and plants. But, owing to the coevolution of pathogens and host interaction, bacterial species have developed their resistance mechanisms to sense and create an adaptive response against AMPs as well, which are playing a crucial role in their virulence within the host (Baindara et al., 2020). There are many AMPs being examined for anti-infectives in clinical settings (Costa et al., 2019; Divyashree et al., 2020). So, Understanding the various resistance mechanisms used by gram-negative and gram-positive bacteria to sense and combat AMP activities will help determine new strategies of novel therapeutic strategies to govern MDR pathogens. Furthermore, for host defense AMPs, for example, cathelicidins and defensins, could be utilized as potential compounds for host-targeted therapeutics for treating drug-resistant diseases in humans (Aslam et al., 2018). However, bacteria may even coevolve toward AMP-resistance mechanisms, resulting in low resistance. Some examples are reported so far in the case of AMPs that require specific recognition molecules, such as LPS, Lipid A, Lipid I/II, and LptD, on the membrane surface of bacteria. Nisin and polymyxin resistance are well-studied among bacterial mutants with changes in membrane and cell wall composition which is the target site of AMPs (Tresnak and Hackel, 2020). More research will be needed to understand the behavior of resistance development causing the coevolution of drug-resistant bacteria in humans in healthcare facilities (Dijksteel et al., 2021).

## Conclusion and Outlook

Difficulties in the management of resistant microbes are considered as a significant challenge to discover and develop powerful medicines that can be effectively utilized against different resistant microorganisms. Recent years have witnessed a surge in the development of AMPs for antimicrobial therapy. On the other hand, a wide variety of nanoparticles have exhibited outstanding benefits for antimicrobial therapy than that of their corresponding bulk-drug formulations. It is evident from the recent studies that the AMP-decorated nanoparticles and polymers can act as an additively-enhanced combination therapeutic/delivery agent, holding considerable promise for antimicrobial therapy. This success has been obtained in minimizing the side effects and achieving the equal or higher therapeutic effects at a low dose. Nanoparticles not only have effective results on the cellular permeation of antimicrobial agents, but also improve their bioavailability compared with the plain form. Of note, the efficacy of AMP-decorated nanoparticles and polymers against pathogens and drug-resistant pathogens changes according to the physicochemical characteristics of nanoparticles, polymers, and AMPs. However, the majority of these results are from the studies performed at laboratory scale and in animal models. By further studies, the clinical fate of AMP-decorated nanoparticles and polymers should be found. Molecular level understanding of the antimicrobial action of AMP-decorated nanoparticles and polymers can establish a scientific basis to develop novel treatments. It will be assumed that in coming years, we will see great investigations on this field to bring AMP-decorated nanoparticles and polymers into pharmaceutical industry with extensive application in different antimicrobial preparations. AMPs also are appropriate as anti-biofilm agents compared to traditional antibiotics. However, the development of anti-biofilm peptides can be a more delicate process compared to that of AMP inhibitors.

## Author Contributions

SM, SS, and KK wrote the different sections of review. J-YL and FL developed the initial plan and correct the final version. All authors contributed to the article and approved the submitted version.

## Funding

This work was supported by the Natural Sciences and Engineering Research Council (NSERC) Discovery Grant (RGPIN 2018-04070) to J-YL.

## Conflict of Interest

The authors declare that the research was conducted in the absence of any commercial or financial relationships that could be construed as a potential conflict of interest.

## Publisher’s Note

All claims expressed in this article are solely those of the authors and do not necessarily represent those of their affiliated organizations, or those of the publisher, the editors and the reviewers. Any product that may be evaluated in this article, or claim that may be made by its manufacturer, is not guaranteed or endorsed by the publisher.

## Footnotes

^1^ https://aps.unmc.edu/
